# Modeling the Effect of the Biological Control of *Pseudococcus viburni* Signoret (Hemiptera: Pseudococcidae) on Grapevine Leafroll Virus Spread

**DOI:** 10.3390/plants14193043

**Published:** 2025-10-01

**Authors:** Katia Vogt-Geisse, Margarita C. G. Correa, Juan Pablo Gutiérrez-Jara, Kent M. Daane

**Affiliations:** 1 Facultad de Ingeniería y Ciencias, Universidad Adolfo Ibáñez, Santiago 7941169, Chile; katia.vogt@uai.cl; 2 Centro de Investigación de Estudios Avanzados del Maule (CIEAM), Vicerrectoría de Investigación y Postgrado, Universidad Católica del Maule, Talca 3480112, Chile; 3 Department of Environmental Science, Policy and Management, University of California, 1919 Addison St., Berkeley, CA 94720, USA

**Keywords:** virus, spread, entomological vector, GLD, mathematical model

## Abstract

Grapevineleafroll disease (GLD) is one of the more severe and persistent diseases in grapevines worldwide and is caused by several species of grape leafroll-associated viruses (GLRaVs). GLRaVs enter vines mainly by infected plant material or insect vectors. Mealybugs (Hemiptera: Pseudococcidae) are important vectors of GLRaVs and, among them, *Pseudococcus viburni* is the primary *key* vector in many regions. To reduce GLRaV spread, acquiring vines from virus-free certified nurseries, removing infected vines, and controlling insect vectors are crucial control tools. Sustainable mealybug control relies on eco-friendly products, cultural practices that limit mealybug population growth, and biological control by natural enemies. For *P. viburni*, biological control is primarily based on the action of predators and parasitoids, such as *Cryptolaemus montrouzieri* Mulsant and *Acerophagus flavidulus* Brethes, respectively, which will obviously have a different mode of action than chemical insecticides. However, the long-term effect of biological control on GLRaV spread within vineyards has rarely been studied. With the aim of better predicting the impact of biological control on insect vectors, such as mealybugs, we developed a mathematical model to predict the GLRaV spread. The results highlight the importance of establishing vineyards with virus-free material and having a pest management program that reduces the vector population to reduce the economic loss from GLRaVs.

## 1. Introduction

Viruses constitute one of the main causal agents of plant diseases and improving their management is critical, especially for those virus species that do not have direct preventive or curative measures [1]. Viruses can be transmitted between plants through unassisted pathways (e.g., through pollen, contact, water, etc.) and assisted pathways (e.g., mites, insects, human management, etc.) [2]. With climate changes, such as global warming, the life cycles and geographic ranges of arthropod vectors will also change, which, in turn, may impact plant pathogen transmission and disease incidence [3]. Grapes are a global crop, with vineyards often planted in regions that are at the vines’ climatic range tolerances, which makes any change in temperature especially relevant [4,5,6]. Moreover, with increased plant trade, there are more shared pests and diseases, as well as guidelines for their control [7].

Wine production is one of the oldest agronomic activities, in fact, the first viticultural records date back to 8000 BC [8]. Grapevine leafroll-associated viruses (GLRaVs) are one of the greatest threats to vineyards worldwide [9]. This complex of ampeloviruses is responsible for grapevine leafroll disease (GLD), which is present in nearly all regions where vineyards are commercially cultivated [10]. For grape growers, the presence of GLD can cause a reduction of up to 40% yield and affect wine quality primarily due to delayed maturation [11,12]. The economic impact of GLD on a vineyard’s lifespan was estimated at $32,500 (USD) per hectare, even with a low incidence of GLRaVs [13].

Initially, grafting was considered to be the main transmission pathway of GLRVs [14]; however, since 1990, the transmission of GLRaVs by scale insects, especially mealybugs (Hemiptera: Pseudococcidae), has been shown to be the primary transmission pathway for GLRaV-1 and GLRaV-3 [15]. Mealybugs appear to have a semi-persistent and non-circulative mode of transmission, acquiring and transmitting the virus within an hour after feeding [16,17]. The estimated transmission probability of a single mealybug, feeding for only a day, varied from 0.1 to 0.4 [18]. Although all mealybug larval stages can transmit GLRaVs, first or second instars are the most efficient [19] and the most mobile [20].

*Pseudococcus viburni* is believed to be native to South America, but is now found worldwide [21]. It has also been shown to transmit GLRaV-3 [22], the most prevalent GLRaV species, and it is suspected that it can transmit other insect-transmissible GLRaVs. Synthetic insecticides have been the most widely used tools for controlling vineyard mealybugs [23]. However, most insecticide applications do not kill all of the mealybugs because some are located in protected areas of the vine (e.g., under the bark). Moreover, there can be non-targeted effects of insecticides on beneficial insects, the potential for insecticide resistance, and environmental health concerns [24]. For these reasons, insecticides are being supplemented or replaced by more sustainable methods, such as biological controls and mating disruption, to meet the consumer demand for more sustainably grown food [7]. In New Zealand, for example, *P. viburni* was brought under exceptional control by the release of the parasitoid *Acerophagus maculipennis Signoret* [25]. Also, the specific gregarious endoparasitoid *Acerophagus flavidulus* has been mainly used in several countries as the primary parasitoid against *P. viburni* [7,26] and the generalist coccidophagous coccinellid, *Cryptolaemus montrouzieri*, has been released as the main predator for mealybug control [27,28,29,30].

Controlling GLD requires an integrated response to maintain a healthy vineyard [31]. Unlike mealybugs and many other arthropod pests, GLRaVs are more difficult to sample and their presence is often diminished or overlooked by grape producers until the virus has spread to a significant portion of the vineyard [9]. For this reason, vineyard managers are often initially concerned with mealybug control—the pest that they can more easily see and sample. The biological control of *P. viburni*, as an example, relies primarily on predators that can interact or compete, which might lead to intraguild predation or interspecific competition, incorporating ecological forces that can interfere with the success of biological control [32,33]. Thus, one aspect of implementing an Integrated Pest Management (IPM) program in vineyards is to maximize the synergistic activities of combinations of natural enemy species, as well as other vineyard management practices that could impact the performance of pests or natural enemies. Therefore, an IPM strategy to control viruses and mealybugs in vineyards must consider the interactions between all the factors involved: plant, insect vector, and viruses.

Here, we investigate aspects of GLD control by modeling the integration of *P. viburni* biological control in vineyards using Impulsive Differential Equations (IDEs). Modeling a pest’s population dynamics through these types of equations involves sudden perturbations or abrupt changes in certain instances during underlying continuous dynamics, which are represented by pulses. These models have been used to describe problems in physics, ecology, biological systems, and epidemiology, among others [34,35,36,37,38]. In particular, they have also been used to model pests that affect plants on agricultural land, incorporating biological control [37,38,39]. Regarding the dynamics of pathogens transmitted by vectors (pests), SIR (susceptible, infectious, removed) or SEIR (susceptible, exposed, infectious, removed) models have been applied, depending on the type of disease and the type of plant affected [40,41,42,43,44,45]. In addition, IDEs based on Lotka–Volterra systems can also be found in the literature, such as models presented in Tang et al. [46] about general pest management and Shi et al. [47] for IPM with disease in the prey and pulse release of an infective prey population, such that predation occurs according to a Holling type II functional response. The effect of the biological control of *P. viburni* on GLRaV contamination and spread in vineyards has not been studied. In this work, we used a mathematical model that describes interactions among vines, mealybugs, and natural enemies, to help determine the release timing of biological control agents to suppress *P. viburni* populations and, in turn, prevent the spread of GLD.

## 2. Results

**Figure 1:**
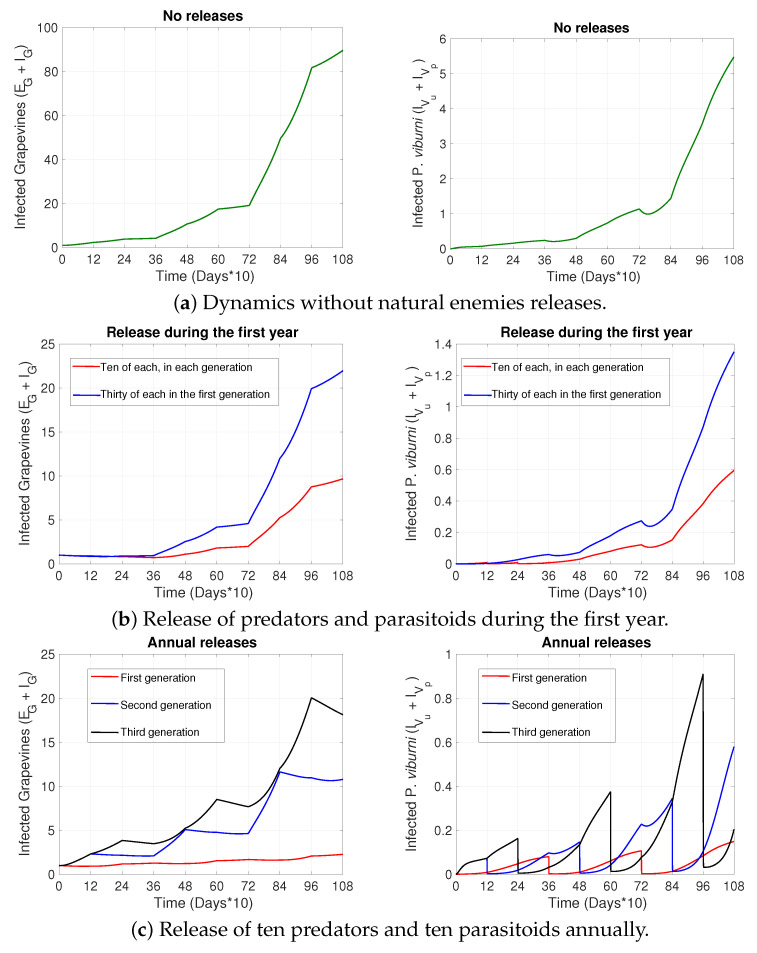
The figure shows the population of infected grapevines ($E_{G}+I_{G}$, left) and infected *P. viburni* ($I_{V_{u}}+I_{V_{p}}$, right) with respect to time: (**a**) depicts the dynamics without control measures; (**b**) in red shows a scenario releasing, during the first year, 10 of both natural enemies at the beginning of each *P. viburni* generation (3×), and in blue a scenario releasing, during the first year, 30 of each only at the beginning of the first *P. viburni* generation; (**c**) shows the dynamics when releasing 10 of both natural enemies every year at the beginning of the first generation (red curve), the second generation (blue curve), and the third generation (black curve). The variables referred to in the legend are defined in Table 1 and the parameters used are as in Table 2, Table 3 and Table 4. $S_{G}\left(0\right)=3999$, $E_{G}\left(0\right)=0$, $I_{G}\left(0\right)=1$, $R_{G}\left(0\right)=0$, $S_{V_{u}}\left(0\right)=10$, $I_{V_{u}}\left(0\right)=S_{V_{p}}\left(0\right)=I_{V_{p}}\left(0\right)=F_{A}\left(0\right)=C_{l}\left(0\right)=C_{A}\left(0\right)=0$.

The dynamics of infected grapevine ($E_{G}+I_{G}$) and infected *P. viburni* ($I_{V_{u}}+I_{V_{p}}$) in the presence of GLD under different biological control scenarios are depicted in Figure 1, under the presence of susceptible mealybugs and initially one infected vine. Specifically, Figure 1a shows the natural dynamics of infected grapevines and vectors without any control measures. Figure 1b depicts the dynamics for infected grapevines and vectors under two natural enemy release scenarios applied only during the first year (0–360 days) after detecting *P. viburni*: (1) in red, ten of both natural enemies are released during the first year of infestation at the beginning of each of the three *P. viburni* generations ($t_{n}=0,120,240;t_{z}=0,120,240$); (2) in blue, thirty of each natural enemy are released but only at the beginning of the first generation of *P. viburni* ($t_{n}=0$, $t_{z}=0$). Figure 1c shows three dynamics for infected grapevines and vectors under the scenario that ten of both natural enemies are released annually in the course of three years (0–360, 360–720, 720–1080 days) but only during the first generation of *P. viburni* (red), the second generation of *P. viburni* (blue), or the third generation of *P. viburni* (black). When releasing natural enemies of *P. viburni*, one can observe in Figure 1— especially in the right subfigure (c)— the changes in the population of infected *P. viburni* due to the pulse implementation of biological control, i.e., each time a generation of *P. viburni* is controlled by natural enemies, the mealybug population reduces noticeably.

There is a considerable difference (∼60%) in the total number of infected grapevines after the release of natural enemies (predators and parasitoids) in a single generation and when distributing that amount among the three generations (see Figure 1b on the left). This decreasing trend is maintained toward reducing infected vectors (see Figure 1b on the right). In terms of release efficiency between generations, it is clear from Figure 1c that the release during the first generation is more efficient than during the next two generations in reducing infected vines and infected vectors, with a more significant long-term impact on the reduction of infected vines.

**Figure 2:**
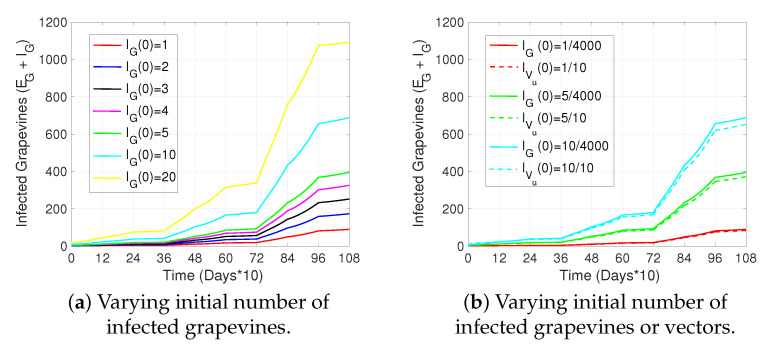
The figure shows the population of infected grapevines ($E_{G}+I_{G}$) with respect to time for different initial conditions: (**a**) depicts the dynamics for varying initial number of infectious vines ($I_{G}\left(0\right)$), while (**b**) for varying initial number of infectious vines ($I_{G}\left(0\right)$, solid curves) or infectious vectors ($I_{V}\left(0\right)$, dashed curves). The variables referred to in the legend are defined in Table 1 and the parameters used are found in Table 2, Table 3 and Table 4.

Figure 2a shows the number of infected grapevines ($E_{G}+I_{G}$) for different initial amounts of infectious grapevines ($I_{G}\left(0\right)$) and initially only susceptible mealybugs. Figure 2b on the other hand compares scenarios where initially there is a certain number of only infectious grapevines ($I_{G}\left(0\right)$, solid lines) vs. of only infectious mealybugs ($I_{V}\left(0\right)$, dashed lines). In particular, in Figure 2b, one can observe very similar dynamics for a small number of initially infectious vines vs. a significant number of initially infectious vectors (compare solid lines and dashed lines of the same color). In general, Figure 2 shows an increasing trend in point prevalence for an increasing initial number of infectious agents.

## 3. Discussion

We have proposed a mathematical model of impulsive differential equations to describe the dynamics of GLRVs between grapevines, *P. viburni* as a vector, and vector control through manipulating natural enemies: *C. montrouzieri* as predators and *A. flavidulus* as parasitoids. The model can serve as input for decision-making on policies for the control of the virus. Moreover, our results provide novel insights to guide biological control agent releases against *P. viburni* in vineyards to reduce the transmission of GLRaVs among grapevines. While still preliminary, these results show how such models can be used to reduce vineyard production losses and management costs, especially those associated with insect pest control, low yield, low wine quality, and infected grapevine replacement.

The IDE model also provides discussion points on the impact of different control strategies of GLRaVs in grapevines through the release of natural enemies against the mealybug vectors. The model suggests that, over a three year period, the most effective way to reduce GLRaV spread and control the vector without pesticides may be to implement annual releases at the beginning of the first generation of *P. viburni* of each year (see Figure 1c, red curve), instead of releasing natural enemies during future generations of mealybugs. Furthermore, releasing predators and parasitoids each year instead of only during the first year may be crucial for control, but only if the yearly release is carried out targeting early generations of mealybugs, that is, the first or second generation (see Figure 1b vs. Figure 1c). More specifically, if control is implemented late each year, while targeting only third generations of mealybugs, pathogen control may be poorer than when releasing natural enemies only during the first year targeting each generation of mealybugs (see Figure 1c black curve vs. red curve). This emphasizes the importance of early yearly detection and control of *P. viburni* to effectively control the disease. The high fecundity of *P. viburni* coupled with cryptic biology could explain the importance of reducing population levels early in the season before it is too late [48]. Even if the biology of *P. viburni* had been studied in the past, the effect of climate change on *P. viburni* population dynamics and grapevine virus transmission warrants further study [49].

On the other hand, our model suggests that preventing the introduction of infected grapevines is also crucial to reducing GLRaV spread. We can observe that a small percentage of initially infected grapevines can have a significant impact on disease prevalence over time, often more than the impact of infected mealybugs entering a vineyard—See for instance the red curves in Figure 2b, where only a 0.025% initial vine infection produces a nearly equivalent incidence curve as the later invasion of 10% infected mealybugs. Nevertheless, infected *P. viburni* entering a vineyard may still produce a significant increase in disease prevalence in grapevines that cannot be ignored (see dashed curves in Figure 2b) and that may be much higher compared to the prevalence observed over time in some cases with initial incidence of grapevines (see Figure 2a vs. dashed curves in Figure 2b). Indeed, even if the virus transmission of GLRVs among plants in the vineyard is mainly carried out by insect vectors, infected plants play a key role as a virus source [50].

Here, we focused on natural enemies for vector suppression. Equally, if not more important, is the removal (roguing) of GLRaV-infected vines [51,52]. Roguing may not always be successful, as GLD has been shown to spread in a replanted vineyard from a preceding vineyard [9] and GLRaV-3 was shown to remain in the roots of removed vines [53]. The population level of mealybugs and leafroll will also impact the effectiveness of roguing, and vineyards with >20% GLRaV-infected vines are often considered to have too much inoculum to effectively treat [31]. Although more information is needed to fully understand the impact of different management scenarios for GLRaV and vector populations on GLD management [54], the results of our IDE model offer a tool that may help predict more sustainable management combinations to improve GLD control in vineyards. For grape growers, the model presented in this work helps to highlight the importance of different strategies for the prevention and control of GLD transmitted by *P. viburni* when facing different scenarios and the relevance of in-time decision-making (i.e., to establish vineyards with certified virus-free vines, to integrate early mealybug control to prevent virus spread, etc.).

The model presented via IDE did not consider environmental, climatic, or economic factors, among others, that could affect the dynamics of disease transmission in grapevines. These factors could enhance research results, so they are left for future work on the transmission and control of grape leafroll-associated viruses (GLRaVs).

## 4. Materials and Methods

### 4.1. The Model

We present a model that illustrates the dynamics of GLRaV dispersion in vineyards with *P. viburni*—the main vector of the disease—present and subjected to different control elements, such as natural enemies. The model includes parameter values that incorporate different seasonal generations of *P. viburni* population dynamics. For example, during winter, mealybugs hide below the soil and under the bark of the vine to lay eggs [48], and then, in spring, the first generation (q=1) of *P. viburni* climbs the vine and commonly resides underneath the sprouts, where it can be transported through the wind, field crews, and equipment to neighboring grapevines, as has been shown for other mealybug species [55]. This first generation lays eggs on the leaves, giving rise to a second generation (q=2) of mealybugs in summer, which subsequently produces the third generation (q=3) that invades the grape cluster at the end of summer. These last two generations can be transported to other grapevines by summer pruning (manual or mechanical) of the vines to reduce foliage. The latter generation then migrates back to the soil and cornices to spend the winter. Of course, this seasonal description could change with regional climatic differences and vine conditions.

GLRaVs may enter the vineyard through infected vegetative material that is planted in the vineyard or through infected grafts, and can be transmitted from grapevines to grapevines through infected mealybugs. Weeds that harbor mealybugs represent an uncontrolled or at least untargeted reservoir of vectors that can later spread GLRaVs when moving to the vines. Therefore, to control virus transmission, it is crucial not only to control the mealybug population, but also to practice sound viticulture practices, such as planting virus-free material [56]. Finally, our model includes the release of natural enemies of *P. viburni* to control the pest, and we study its effect on the control of the virus, together with the effect of human risk perception on the presence of the virus.

### 4.2. Model Description

**Figure 3:**
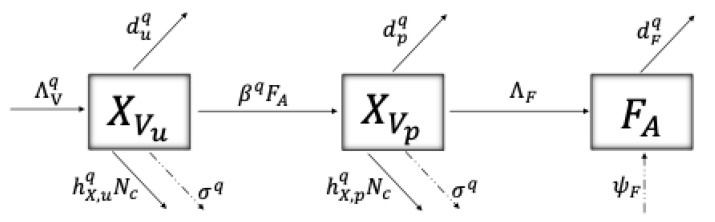
The schematic shows the flow between the states of unparasitized ($X_{V_{u}}$) and parasitized ($X_{V_{p}}$) populations of *P. viburini*, and the population of the parasite *A. flavidulus* ($F_{A}$). The solid arrows represent transitions rates between compartments (recruitment rate ($\Lambda_{V}^{q}$), parasitism rate ($\beta^{q}F_{A}$), parasite hatching rate ($\Lambda_{F}$)), and continuous exit rates of each population through death ($d_{u}^{q}$, $d_{p}^{q}$, $d_{F}^{q}$) or predation of *P. viburni* ($h_{X,u}^{q}N_{c}$, $h_{X,p}^{q}N_{c}$) that occurs by the population $N_{c}$ of *C. montrouzieri*, whereas the dashed–dotted–dashed arrow represents the pulse release of *A. flavidulus* into the system for the biological control of *P. viburni* modeled by an incremental factor $\psi_{F}$ that increases the population of *A. falvidulus* at certain instants $t_{z}$ (see System (5)). The dashed–dotted–dotted arrows stand for an incremental factor ($\sigma^{q}$) of the vector mortality rate, which occurs at certain instants $t_{m}$ (see System (5)) due to the implementation of preventive and vector control measures. Table 2 describes the parameters used and System ((5) shows this schematic translated into impulsive differential equations).

Figure 3 shows the dynamics of the biological process of *P. viburni* considered in the model. Table 1 describes the variables in the model. In particular, $X_{V_{u}}$ and $X_{V_{p}}$ represent unparasitized and parasitized *P. viburni*, respectively, where *X* stands for two possible epidemiological stages of *P. viburni*: X=S, susceptible (has not aquired the virus), or X=I, infectious (acquired the virus and is able to transmit GLD to grapevines). The recruitment rate of *P. viburni* is into the unparasitized population and is given by $\Lambda_{V}^{q}$. $\beta^{q}$ corresponds to the parasitism rate of each *Acerophagus flavidulus* ($F_{A}$) when encountering an unparasitized mealybug, and hence the parasitism rate per mealybug is given by $\beta^{q}F_{A}$. *A. flavidulus* ($F_{A}$) hatches from its host at a rate $\Lambda_{F}$, entering the class $F_{A}$ of adult *A. flavidulus*.

**Table 1:** Description of the variables of the model in System (5).

Variable	Description	Units
*P. viburni*		
$X_{V_{k}}\left(t\right)$	Susceptible (X=S) or infectious (X=I) state at development	v
	stage $V_{k}$, k=u,p	
$X_{V_{u}}\left(t\right)$	Unparasitized population of *P. viburni* at time *t*	v
$X_{V_{p}}\left(t\right)$	Parasitized population of *P. viburni* at time *t*	v
*A. flavidulus*		
$F_{A}\left(t\right)$	Population at time *t*	f
*C. montrouzieri*		
$C_{l}\left(t\right)$	Population at larval stage, at time *t*	m
$C_{A}$	Population at adult stage, at time *t*	m
*Grapevines*		
$S_{G}\left(t\right)$	Susceptible grapevines at time *t*	g
$E_{G}\left(t\right)$	Exposed grapevines at time *t*	g
$I_{G}\left(t\right)$	Infectious grapevines at time *t*	g
$R_{G}\left(t\right)$	Removed (uprooted) grapevines at time *t*	g

v = *P. viburni*, f = *A. flavidulus*, m = *C. montrouzieri*, g = grapevines.

*P. viburni* are preyed upon by *C. montrouzieri*, whose total population is represented by $N_{C}=C_{l}+C_{A}$, which includes larval- ($C_{l}$) and adult-stage ($C_{A}$) predators. Hence, due to predation, *P. viburni* leave the $X_{V_{k}},\;k\in \left\{u,p\right\}$ classes according to a predation rate that is modeled by the following functional response:

(1) $$h_{X,k}^{q}=p_{k}^{q}\frac{X_{V_{k}}}{K^{q}+X_{V_{k}}},\;k\in \left\{u,p\right\},\;X\in \left\{S,I\right\}\;$$

where $p_{k}^{q}$ expresses the preference for predation for unparasitized (k=u) and parasitized (k=p) vectors, and $K^{q}$ corresponds to a constant. The natural mortality rate of *P. viburni* is represented by $d_{k}^{q}$, k∈{u,p}, and the one of *A. flavidulus* by $d_{F}$. Also, at certain times (pulses at instants $t_{m}$), prevention and control measures are applied to vectors, leading to an increase in the vector mortality rate, denoted by an incremental factor $\sigma^{q}$. Finally, *A. flavidulus* is pulse-released (instants $t_{z}$), increasing the parasitoid population by an incremental factor of $\psi_{F}$.

**Figure 4:**
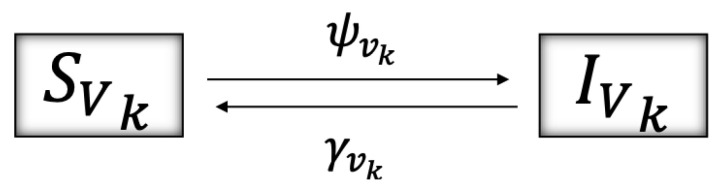
Schematics of the virus acquisition dynamics of *P. viburni*, where susceptible unparasitized and parasitized *P. viburni* ($S_{V_{k}}$, k=u,p) when feeding from an infected grapevine with probability ($I_{G}/N_{G}$), acquire the virus according to the force of infection given by $\psi_{v_{k}}=\frac{\beta_{v}^{q}}{r_{k}^{2}}S_{V_{k}}I_{G}/N_{G}$ (see Equation (2)), moving to the infectious stage $I_{V_{k}}$, k=u,p, at which *P. viburni* are capable of transmitting the virus to grapevines. *P. viburni* lose infectiousness and return to the susceptible class according to a rate $\gamma_{v_{k}}$. Table 2 describes the parameters used and System (5) shows this schematic translated into differential equations.

As mentioned before, the *P. viburni* population follows virus-acquiring dynamics—represented in Figure 4—such that $X_{V_{k}}$, k=u,p, with X=S, represent the susceptible (virus-free, susceptible to acquire the virus) and X=I the infectious (virus acquired, able to infect grapevines) epidemiological stages of *P. viburni*.

**Table 2:** Description of parameters related to *P. viburni* from the model in System (5).

Param.	Description	Units	Baseline	Value	Ref.
$\Lambda_{V}^{q}$	Recruitment rate.	${\mathrm{vd}}^{-1}$	$\left[\frac{1}{30},\frac{1}{2}\right]$	$\sum_{k}d_{k}^{q}$	[57]
$\beta^{q}$	Parasitation rate of *P. viburni* by *A. flavidulus*.	$d^{-1}f^{-1}$	$\left[\frac{1}{10},1\right]$	0.5	[58]
$d_{u}^{q}$, $d_{p}^{q}$	Natural mortality rate.	$d^{-1}$	$\left[\frac{1}{22},\frac{1}{2}\right]$	1/30, 1/30	[58]
$p_{u}^{q},p_{p}^{q}$	Predator preference proportion for unparasitized, parasitized *P. viburni*,	Ul	[0,1]	0.6, 0.4	[38]
$\sigma^{q}$	Mortality incremental factor due to vector prevention and control measures differentiated by season q=1,2,3.	Ul	[1,∞[	3/10, 3/100, 0	[58]
$K^{q}$	Constant in the functional response of the predation rate.		[1,∞[	1000	[58]
$\beta_{v}^{q}$	Transmission rate from infectious vine to susceptible vector.	$d^{-1}$		65/100	
$1/r_{k}^{2}$	Vector–host transmission rate reduction due to vector movement.	Ul	]0,1]	1/4	[40,59,60]
$1/\gamma_{v_{k}}$	Average time a vector remains infectious.	d	]0,16]	10	[17,61]

d = day, v = *P. viburni*, f = *A. flavidulus*, Ul = Unitless.

The force of infection for a vector in class $S_{V_{k}}$, k=u,p, to acquire the virus and become infectious is given by

(2) $$\psi_{V_{k}}=\frac{\beta_{v}^{q}}{r_{k}^{2}}S_{V_{k}}I_{G}/N_{G},$$

where $\beta_{v}^{q}/r_{k}^{2}$ is the infection rate for a susceptible vector when feeding on an infectious vine with probability $I_{G}/N_{G}$, where $I_{G}$ represents the population of infectious vines and $N_{G}$ the total number of grapevines. The form of the infection rate can be interpreted as following a baseline transmission rate $\beta_{v}^{q}$, which is reduced due to vector movement ($r_{k}^{2}$). An infectious vector loses its infectiousness and returns to the susceptible class according to the rate $\gamma_{v_{k}}$. Table 2 describes the parameters of the model related to *P. viburni*’s biological and virus acquisition dynamics.

**Figure 5:**
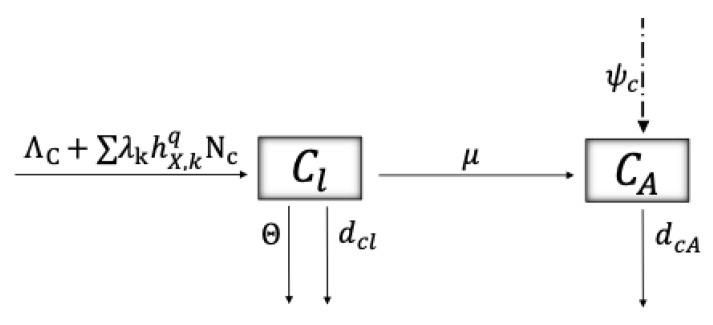
The schematic shows the flow between the larval stage ($C_{l}$) and the adult stage ($C_{A}$) of *C. montrouzieri*. The solid arrows represent transition rates between compartments (recruitment rate ( $\Lambda_{C}$), predator benefit rate when feeding on *P. viburni* ($\sum_{k=u,p}\lambda_{k}h_{X,k}^{q}N_{C}$), transition rate from larvae to adult (μ), natural death rates ($d_{cl},\;d_{cA}$), and death rate due to cannibalism (Θ)), whereas the dashed–dotted–dashed arrow represents the pulse release of *C. montrouzieri* at instants $t_{n}$ into the system, modeled by an incremental factor $\psi_{C}$ that increases the population of adult *C. montrouzieri* for the biological control of *P. viburni*. Table 3 describes the parameters of the model related to *C. montrouzieri*, and System (5) translates the schematic to impulsive differential equations.

The dynamics of the population of the predator *C. montrouzieri* are shown in Figure 5, where–as mentioned before– $C_{l}$ and $C_{A}$ represent *C. montrouzieri* in the larval and adult stages, respectively, and $N_{C}=C_{l}+C_{A}$ their total population. The average time that a ladybug remains in the larval stage before becoming an adult is 1/μ. $\Lambda_{C}$ corresponds to the natural recruitment rate as larvae and $\sum_{k=u,p}\lambda_{k}h_{X,k}^{q}N_{C}$ represents the benefit to the *C. montriouzieri* population after predation on *P. viburni*. $d_{cl}$ and $d_{cA}$ are the natural mortality rates of the larval and adult predator stages, respectively. Θ represents the mortality rate due to cannibalism after predator overpopulation and is defined by $\Theta :=\theta [N_{C}/\bar{C}]$, where [·] is the integer part function and $\bar{C}$ the carrying capacity of the *C. montrouzieri* population. Finally, the release of natural predators at certain instants (pulses at instants $t_{n}$) is represented by the dashed–dotted–dashed line, and represented by an incremental factor of the adult population $\psi_{C}$. Table 3 describes the parameters related to the dynamics of parasitoids and predators.

**Figure 6:**
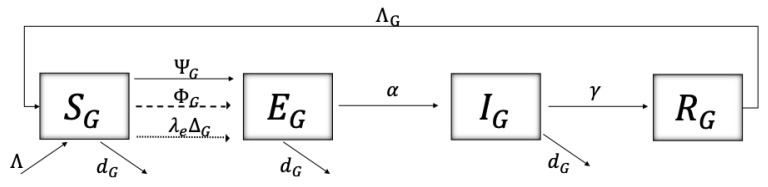
Schematics of the disease dynamics for vines, where the population is divided into susceptible ($S_{G}$), exposed (infected but not yet infectious) ($E_{G}$), infectious ($I_{G}$), and removed ($R_{G}$) vines. Susceptible vines can acquire the virus through infected *P. viburni* at a force of infection $\Psi_{G}$, through pruning with infected equipment at instants $t_{j}$ and at a force of infection $\Phi_{G}$, or through infected grafting at instants $t_{i}$ at a rate $\lambda_{e}\Lambda_{G}$. Exposed vines transition to being infectious at a rate α and infectious vines are removed at a rate γ. Removed vines get replaced by susceptible vines at a rate $\Lambda_{G}$. All, except removed vines, have a natural death rate $d_{G}$, and grapevines are recruited as susceptible vines at a rate Λ. Table 4 describes the parameters of the model related to grapevines, and System (5) translates the schematic to impulsive differential equations.

Finally, the dynamics between epidemiological states of the grapevine population are shown in Figure 6. The population is divided into susceptible vines ($S_{G}$), exposed (infected but not yet infectious) vines ($E_{G}$), infectious vines ($I_{G}$) (which we assume to be symtomatic), and removed vines ($R_{G}$). The transition from susceptible grapevines ($S_{G}$) to exposed grapevines ($E_{G}$) can occur in three different ways:

**Table 3:** Description of parameters related to *A. flavidulus* and *C. montrouzieri* from the model in System (5).

Param.	Description	Units	Baseline	Value	Ref.
*A. flavidulus*					
$\Lambda_{F}$	Recruitment rate.	$d^{-1}$	–	1/10	[48]
$d_{F}$	Natural mortality rate.	$d^{-1}$	$\left[\frac{1}{12},\frac{1}{8}\right]$	0.1	[48]
$\psi_{F}$	Release incremental factor at instants $t_{z}$.	Ul	-	$\frac{Number\;released}{F_{A}\left(t_{z}\right)}$	CA
*C. montrouzieri*					
$\Lambda_{C}$	Recruitment rate.	${\mathrm{md}}^{-1}$	$\left[\frac{1}{14},\frac{1}{7}\right]$	0.1	[48]
$\lambda_{k}$	Predation conversion rate from *P. viburni* to *C. montrouzieri*.	$d^{-1}$	[0,1]	0.001	[48]
θ	Cannibalism rate.	$d^{-1}$	[0,1]	1	[38]
1/μ	Mean larval stage duration before becoming adult.	d	[20,30]	25	[48]
$d_{cA}$	Natural mortality rate of adults.	$d^{-1}$	$\left[\frac{1}{50},\frac{1}{40}\right]$	0.02	[48]
$d_{cl}$	Natural mortality rate of larvae.	$d^{-1}$	$\frac{4}{100}\times \left[\frac{1}{50},\frac{1}{40}\right]$	$\frac{4}{100}\times d_{cA}$	[48]
$\psi_{C}$	Release incremental factor at instants $t_{n}$.	Ul	-	$\frac{Number\;released}{C_{A}\left(t_{n}\right)}$	CA
k∈{u,p}. d = day, m = *C. montrouzieri*, Ul = Unitless, CA = Chosen by author					

(i) through infected *P. viburni* ($I_{V_{k}}$, k=u,p) when feeding on susceptible vines ($S_{G}$) with probability $S_{G}/N_{G}$, according to a force of transmission

(3) $$\Psi_{G}=\sum_{k=u,p}\frac{\beta_{G}^{V_{k},q}}{r_{k}^{2}}I_{V_{k}}\frac{S_{G}}{N_{G}},\;$$

where $\beta_{G}^{V_{k},q}/r_{k}^{2}$ represents the transmission rate, whose form is dependent on a base line transmission rate $\beta_{G}^{V_{k,q}}$, which is dependent on the seasonal generations of *P. viburni* (q=1,2,3) and reduced by the movement of the vector ($r_{k}^{2}$).

(ii) through pruning with infected equipment, which occurs at certain instants (pulses at instants $t_{j}$, dashed line), at a force of transmission

(4) $$\Phi_{G}=\beta_{G}^{P,q}S_{G}\frac{I_{G}}{N_{G}},\;$$

where $\beta_{G}^{P,q}$ represents the transmission rate of a susceptible vine ($S_{G}$), that came in contact through pruning with infected vines with probability $I_{G}/N_{G}$, and which is dependent on the seasonal generations of *P. viburni* (q=1,2,3).

(iii) through infected grafting in vineyards that occurs at certain instants (pulses at instants $t_{i}$, dotted line) and at a rate $\lambda_{e}\Delta_{G},$ where $\Delta_{G}$ is the rate of introduction of the grafts and $\lambda_{e}$ represents the proportion of infected grafts.

A grapevine leaves the exposed class ($E_{G}$) by moving at a rate α to the infectious class $I_{G}$. Vines in the latter class can be removed from the vineyard at a rate γ entering the removed class $R_{G}$. The time that it takes for removed grapevines to be replaced by susceptible vines is given by $1/\Lambda_{G}$ and the mortality rate of all except the removed vines is given by $d_{G}$. Table 4 describes the parameters related to grapevines. The model is presented by the system of impulsive differential equations given in (5).

**Table 4:** Description of parameters related to grapevines from the model in System (5).

Param.	Description	Units	Baseline	Value	Ref.
$\beta_{G}^{V_{k},q}$	Transmission rate to grapevine through *P. viburni* at both of its *k*th stages (k=u,p), differentiated by seasonal generations of *P. viburni* q=1,2,3	$d^{-1}$	[0,1]	1, 1/2, 1/8	[18]
$\beta_{G}^{P,q}$	Transmission rate to grapevine through pruning, differentiated by seasonal generations of *P. viburni* q=1,2,3	$d^{-1}$	[0,0.01]	0.01 × 0, 0.01 × 1/3, 0.01 × 1	[37]
1/α	Average virus incubation time	d	[30,180]	180	[37]
1/γ	Average time an infectious vine is uprooted	d	[360,1080]	720	[37]
$\Lambda_{G}$	Replacement rate of removed grapevines.	$d^{-1}$	[0,1/180]	1/360	[62]
Λ	Recruitment rate	${\mathrm{g d}}^{-1}$	$N_{G}\times d_{G},N_{G}=1$	1/9000	
$\Delta_{G}$	Grafting rate	$d^{-1}$	[0,1]	0	[63,64]
$\lambda_{e}$	Proportion of infected grafts	Ul	[0,1]	0	[37]
$d_{G}$	Natural mortality rate	$d^{-1}$	[0,0.02]	1/9000	[13]
$t_{i}$	Time instants where grafting occurs	d	[0,108]	0	[63,64]
$t_{j}$	Time instants where pruning occurs	d	[0,108]	120+360w, 480+360w, w=1,2.	[63,64]

d = day, g = *grapevine*, CA = Chosen by author.




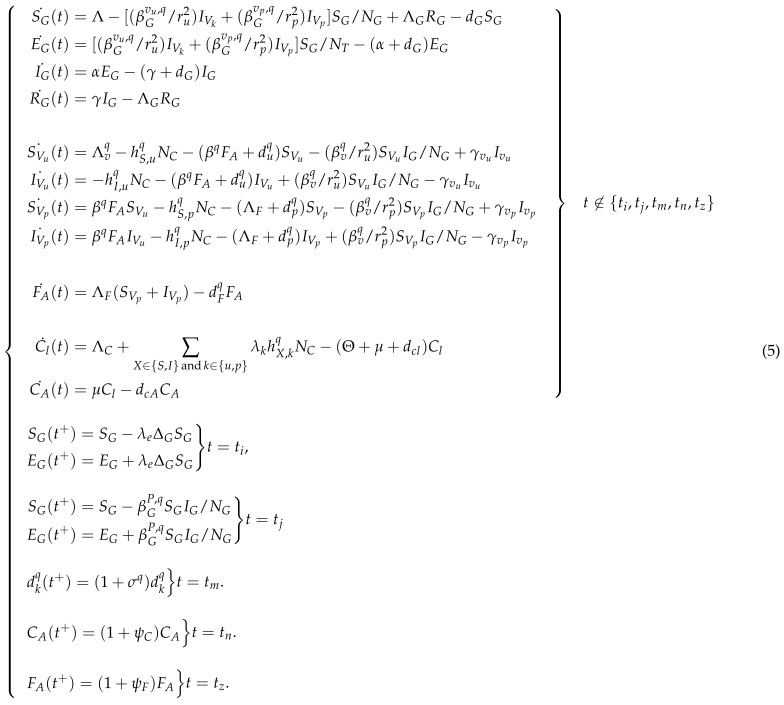




## Data Availability

The data presented in this study are available on request from the corresponding author.
